# DNA Repair and Cell Cycle Biomarkers of Radiation Exposure and Inflammation Stress in Human Blood

**DOI:** 10.1371/journal.pone.0048619

**Published:** 2012-11-07

**Authors:** Helen Budworth, Antoine M. Snijders, Francesco Marchetti, Brandon Mannion, Sandhya Bhatnagar, Ely Kwoh, Yuande Tan, Shan X. Wang, William F. Blakely, Matthew Coleman, Leif Peterson, Andrew J. Wyrobek

**Affiliations:** 1 Life Sciences Division, Lawrence Berkeley National Laboratory, Berkeley, California, United States of America; 2 Center for Biostatistics, The Methodist Hospital Research Institute, Houston, Texas, United States of America; 3 Department of Materials Science and Engineering, Department of Electrical Engineering, Stanford University, Stanford, California, United States of America; 4 Armed Forces Radiobiology Research Institute, Uniformed Services University of the Health Sciences, Bethesda, Maryland, United States of America; 5 Radiation Oncology, UC Davis School of Medicine, University of California Davis, Davis, California, United States of America; University Medical Center Hamburg-Eppendorf, Germany

## Abstract

DNA damage and repair are hallmarks of cellular responses to ionizing radiation. We hypothesized that monitoring the expression of DNA repair-associated genes would enhance the detection of individuals exposed to radiation versus other forms of physiological stress. We employed the human blood *ex vivo* radiation model to investigate the expression responses of DNA repair genes in repeated blood samples from healthy, non-smoking men and women exposed to 2 Gy of X-rays in the context of inflammation stress mimicked by the bacterial endotoxin lipopolysaccharide (LPS). Radiation exposure significantly modulated the transcript expression of 12 genes of 40 tested (2.2E-06<p<0.03), of which 8 showed no overlap between unirradiated and irradiated samples (*CDKN1A, FDXR, BBC3, PCNA, GADD45a, XPC, POLH* and *DDB2*). This panel demonstrated excellent dose response discrimination (0.5 to 8 Gy) in an independent human blood *ex vivo* dataset, and 100% accuracy for discriminating patients who received total body radiation. Three genes of this panel (*CDKN1A, FDXR* and *BBC3)* were also highly sensitive to LPS treatment in the absence of radiation exposure, and LPS co-treatment significantly affected their radiation responses. At the protein level, BAX and pCHK2-thr68 were elevated after radiation exposure, but the pCHK2-thr68 response was significantly decreased in the presence of LPS. Our combined panel yields an estimated 4-group accuracy of ∼90% to discriminate between radiation alone, inflammation alone, or combined exposures. Our findings suggest that DNA repair gene expression may be helpful to identify biodosimeters of exposure to radiation, especially within high-complexity exposure scenarios.

## Introduction

Biological markers of exposure to ionizing radiation (IR) in human populations are of great interest for assessing normal tissue injury in radiation oncology and for biodosimetry in nuclear incidents and accidental radiation exposures. Current approaches to radiation biodosimetry include assessments of physical effects, such as time to emesis and blood lymphocyte kinetics, and cellular determinants such as cytogenetic biodosimetry to assess radiation-induced chromosome aberrations in circulating blood lymphocytes [1]. However, these methods are time-consuming and do not provide results fast enough to identify people who would benefit the most from medical intervention immediately after irradiation. The use of biochemical markers, such as changes in transcript or protein expression or posttranslational modifications, represents an alternative method with the potential for high-throughput, deployable methods for initial triage as well as for the estimation of exposure dose (reviewed in [1], [2]).

Recent studies have identified large-scale changes in transcript expression in irradiated blood lymphocytes shortly following IR exposures and that transcript changes can persist for days after exposure [3]–[12]. A 2006 literature review from our laboratory identified over 260 radiation-responsive proteins and ranked them according to their potential usefulness in human biodosimetric applications [13]. Genes involved in cellular DNA damage response and repair functions, including DNA repair, cell cycle functions and apoptosis were identified as priority candidates for radiation biodosimetry.

DNA is a critical cellular target of IR and the ability of the cell to repair DNA damage determines its fate after exposure. Various forms of DNA damage are induced by IR, including DNA-protein cross-links, base and sugar alterations, DNA single-strand breaks (SSBs), bulky lesions (i.e. clusters of base and sugar damage) and double-strand breaks (DSBs) [14]. The immediate response to IR-induced DNA damage is the stimulation of the DNA repair machinery and the activation of cell cycle checkpoints, followed by down-stream cellular responses such as apoptosis that removes damaged cells. The predominant repair pathway is base excision repair (BER), which is responsible for the removal of damaged bases and DNA single-strand breaks through gap-filling by DNA polymerase and ligation of DNA ends [15]. Nucleotide excision repair (NER) is the major pathway for the repair of bulky DNA damages that cause DNA helical distortion [16]. NER proteins are also involved in repair of oxidative damage through stimulation of BER, including XPC and XPG, indicating cross-talk between these two repair pathways [17]–[21]. Several NER genes are upregulated at the gene expression level by IR, including *XPC* and *DDB2* [4], [22]. IR exposure is known to modulate transcript and/or protein levels of several cell cycle regulators (*CDKN1A* (*p21*), *GADD45a, Cyclin G1* (*CCNG1*), *CHK2*-thr68) and apoptosis genes (*BAX* and *BBC3)* in diverse cell and blood model systems (*in vivo*, *in vitro* and *ex vivo)* [6], [8], [10], [23]–[28]. However, little is known of how co-exposure to confounding factors can affect the utility of individual biomarkers for radiation biodosimetry [1], [29], [30].

The human blood *ex vivo* irradiation exposure model has been used to investigate the early radiation-induced biological responses for potential biodosimetry applications, and was recently demonstrated to accurately reflect the *in vivo* peripheral blood radiation response in humans [12]. Our study utilized this model to examine: (i) the transcriptional response of 40 well known DNA repair, cell cycle control and apoptosis genes after exposure to IR; (ii) IR-induced transcript changes associated with changes in a selected set of proteins; and (iii) transcript and protein responses in the context of inflammatory stress. Lipopolysaccharide (LPS), the principal component of the outer membrane of Gram-negative bacteria [31], elicits strong inflammatory responses and induces oxidative stress in exposed mammalian cells [32]. Our findings demonstrate that inflammation significantly confounds the radiation response of some DNA repair genes at a dose that is relevant for radiation biodosimetry. We identified a small panel of DNA repair transcripts and proteins whose expression changes distinguish between unirradiated and 2 Gy *ex vivo* irradiated human blood samples display excellent radiation dose and time dependent responses in an independent *ex vivo* irradiated human dataset, show robust non-overlapping responses in blood samples from human patients treated with total body irradiation, and demonstrate high accuracy for classifying blood samples receiving radiation only, inflammation stress alone, or both.

## Materials and Methods

### Human Subjects

All research involving human subjects were approved by the Lawrence Berkeley National Laboratory Institutional Review Board. Peripheral blood from healthy volunteers was obtained after written informed consent and was drawn into sodium citrate (whole blood culture model) or sodium heparin (PBMC culture model) Vacutainer tubes (Becton Dickinson and Company, Franklin Lakes, NJ).

### Whole Blood *ex vivo* Radiation Model

Five donors (2 male, 3 female; age range, 20–50 years) provided two peripheral blood samples each, at least one month apart for measurement of transcript and protein responses. Blood collected in Vacutainer tubes was transferred in 18 ml aliquots into 50 ml conical tubes. Blood in tubes was exposed at room temperature to 0 or 2 Gy X-rays, (∼780 mGy/min; Pantak 320 kVp X-ray machine (Precision X-ray); run at 300 kV and 10 mA). Dosimetry was performed using a RadCal AccuPro dosimeter by measuring the accumulated dose over a specific time interval. After irradiation, blood samples were diluted 1∶1 with RPMI 1640 medium (Sigma-Aldrich) supplemented with 10% heat-inactivated fetal bovine serum (Invitrogen) in 50 ml centrifuge tubes, loosely capped and maintained on a 10 degree angle at 37°C in a humidified incubator with 5% CO_2_ for 24 hrs. LPS was added to some blood cultures immediately after irradiation (50 ng/ml LPS from *Escherichia coli* O111:B4) (Sigma Aldrich). After 24 hrs, buffy coats were extracted for protein and RNA purification. Plasma was collected, aliquoted and stored at −80°C.

### RNA Isolation and Quantitative RT-PCR

RNA was isolated using Trizol reagent (Invitrogen) and purification was performed according to the manufacturer’s instructions. In brief, cell pellets were homogenized in Trizol reagent (1.2 ml). The lysed cells were incubated for 5 min at room temperature, followed by the addition of 0.25 ml chloroform. After mixing, the samples were centrifuged at 12,000 g for 15 min at 4°C. The aqueous phase was separated and 0.625 ml ice-cold isopropanol was used to precipitate RNA. The samples were incubated at room temperature for 10 min and total RNA was collected by centrifugation at 12,000 g for 10 min at 4°C. The RNA pellet was washed with 1 ml 70% ethanol and dissolved in 40 µl RNase-free deionized water. The RNA was quantified using a NanoDrop-2000c spectrophotometer (Thermo Scientific), and quality was monitored with the Agilent 2100 Bioanalyzer (Agilent Technologies, Santa Clara, CA). RNA integrity numbers (RIN) ranged from between 7 to 9.5 (mean, 8.3), and 260/280 absorbance ratios ranged from 1.7 to 1.95 (mean, 1.86) [33].

For cDNA synthesis, an aliquot of 4 µg of total RNA was reverse transcribed using the High-Capacity cDNA Reverse Transcription Kit (Applied Biosystems, Foster City, CA) according to the manufacturer's instructions. Taqman Gene Expression Assays (Applied Biosystems, Foster City, CA, USA) were used, according to manufacturer’s instructions, to detect mRNA of 40 DNA damage response genes. 13 genes were selected for further investigation (*BAX*, Hs00180269_m1*, *BBC3*, Hs00248075_m1*, *FDXR*, Hs01031624_m1, *CDKN1A*, Hs00355782_m1*, *GADD45a*, Hs99999173_m1, *CCNG1*, Hs00171112_m1*, *CHK2*, Hs00200485_m1*, *PCNA*, Hs00696862_m1, *LIG1*, Hs01553527_m1*, *XPC*, Hs01104206_m1*, *DDB2*, Hs03044953_m1*, *POLH*, Hs00982625_m1*, *RAD51*, Hs00153418_m1*; * indicates manufacturer’s recommended assay for this gene, if more than one assay was available). See **Table S1** for a full list of all 40 genes and assay numbers. The RT-PCR reactions were performed, in individual reaction format, with the ABI 7500 Fast Real Time PCR System using Taqman Fast Universal PCR Master Mix from ABI and following manufacturer's recommendations. The results were expressed as the threshold cycle (Ct), i.e. the cycle number at which the PCR product crosses the threshold of detection. The relative quantification of the target transcripts normalized to the endogenous control *ACTB* (*β*-*Actin*) was determined by the comparative Ct method (ΔCt) according to the manufacturer’s protocol. The endogenous control gene *GAPDH* was run concurrently but was not used for normalization since LPS treatment induced changes in *GAPDH* transcript levels. Relative fold inductions were calculated by the ΔΔC_T_ method [7]. All samples were run in triplicate. A no RT qPCR control was included for all reactions to monitor for genomic DNA contamination and was negative across all reactions.

### Confirmation of Radiation Responsiveness of Our 8-gene Transcript Panel in Independent Expression Datasets of *ex vivo* Irradiated Human Blood and Blood Samples of Human Patients Undergoing total body Irradiation

Globally normalized whole genome microarray expression profiles of whole blood irradiated *ex vivo* were obtained from the NCBI GEO database (GSE8917; [10]). In that study, human peripheral blood was obtained from five donors and irradiated *ex vivo* (sham, 0.5, 2, 5 and 8 Gy). RNA was isolated from samples collected at 6 and 24 hrs after exposure and transcript levels were measured using Agilent-012391 Whole Human Genome Oligo Microarray G4112A [10]. We then mean normalized the expression levels of each of the eight genes in this dataset across doses, times, and donors, and then summed theses values across all 8 genes for each blood sample in each treatment group. For calculating dose response, we plotted the average and standard error for each dose group, normalized to the average value of the sham group for each of the two time points.

Globally normalized whole genome expression profiles of patients undergoing total body irradiation (TBI) were obtained from the NCBI GEO database (GSE20162; [12]). In that study, peripheral blood gene expression profiles were obtained from 18 donors undergoing TBI. Patients were exposed to a total of 3.75 Gy in one day divided into three equal fractions of 1.25 Gy with approximately 4 hrs between fractions. Blood was collected before irradiation, 4 hrs after the first fraction of 1.25 Gy and 20–24 hrs after the first fraction. Blood from 14 healthy donors was collected as control. RNA was extracted and transcript levels were measured using Agilent Whole Human Genome Microarray G4112A [12]. For the TBI dataset, we again mean normalized the expression levels of each of our 8 genes across all samples in the database for the patients and healthy donors. For each blood samples we calculated the sum of the normalized expression of each of the 8 genes, normalized to the average of the sum expression of the healthy donors.

### Peripheral Blood Mononuclear Cell (PBMC) *ex vivo* Radiation Model

Seven donors (6 male, 1 female; age range, 20–50 years) provided two peripheral blood samples each, at least one month apart, for protein analyses. Blood was transferred to 3 equal (∼13 ml) aliquots into 50 ml conical tubes. Blood in tubes was exposed at room temperature to 0, 2 or 6 Gy X-rays at a rate of ∼1.25 Gy/min (2 Gy) or ∼1.30 Gy/min (6 Gy) (X-ray machine (Faxitron) set at 160 kV and 6.3 mA). Dosimetry was performed using a RadCal AccuPro dosimeter by measuring the accumulated dose over a specific time interval. After irradiation, blood samples were separated over Accuspin System-Histopaque-1077 (Sigma-Aldrich) according to the manufacturer’s instructions. PBMC were washed in phosphate buffered saline (PBS) and resuspended in RPMI 1640 medium (Sigma-Aldrich) supplemented with 10% heat-inactivated fetal bovine serum (Invitrogen) and cultured in duplicate T25 flasks for each dose group. Cells were maintained on a rocking platform at 37°C in a humidified incubator with 5% CO_2_ for 6 and 24 hrs.

### Cell Lysates for Protein Analyses by Enzyme-linked Immunosorbent Assays (ELISA)

Buffy coats were collected from whole blood cultures and treated with a 1∶3 mixture of warm (37°C) RBC Lysis buffer (5 Prime) for two steps and then washed once with cold phosphate buffered saline. The obtained cell pellets or PBS-washed PBMC pellets, from the PBMC culture model, were then lysed with Pierce M-PER Mammalian Protein Extraction Reagent and 1X Halt protease/phosphatase inhibitors (Thermo Scientific). Extracts were collected, aliquoted and stored at −80°C. Protein concentrations were measured using Pierce BCA Protein Assay Reagent (Thermo Scientific). Amounts of protein in lysate or plasma were quantified using ELISA kits; human BAX ELISA kit (Assay Designs), human phosphorylated CHK2-thr68 ELISA kit (Cell Signalling). The biological effectiveness of LPS was confirmed by measuring secretion of IL-6 and TNF-α in plasma by ELISA following manufacturer’s recommended protocol (R&D Systems). ELISAs were performed following manufacturer’s instructions. BAX ELISAs were performed using 0.1 or 1 µg protein cell lysate per well for irradiated and non-irradiated samples, respectively. pCHK2-thr68 ELISAs were performed using 25 µg of protein cell lysate for all samples. IL-6 and TNF-α ELISAs were performed using undiluted or 1∶100-fold dilution of plasma for non-LPS and LPS-co-treated samples, respectively. All ELISA absorbance readings were read with reference to the standard curve, except for pCHK2-thr68 that had no standard curve and used average difference data between control and test samples as readout (**Figure S1**). The majority of absorbance readings were within a 0.1–0.7 range (**Table S2**). All samples were run in duplicate. ELISA plates were read using TECAN Infinite M200 plate reader and analyzed using the TECAN Magellan software. Raw ELISA data was log_e_ transformed and mean-zero standardized to obtain a standard normal distribution. We performed inferential tests of hypothesis via independent 2 sample t-tests for each dose-time experimental group for each biomarker.

### Classification Analysis

We investigated the classification characteristics of our panel of transcript and protein biomarkers for assigning individual samples into their correct exposure/treatment group: no treatment (N), radiation exposure only (R), LPS treatment only (L), and samples exposed to both radiation and LPS (RL). Classification was performed using marker expression values for n = 40 observations per marker, based on the replicate pair of observations per subject and 4 classes (40 = 2×5×4). A 4-class problem was considered for which the true class labels of observations were N, R, L, and RL. Marker order was determined via filtering by considering all possible pairs of classes, and for each pair ranking all 9 markers by their Gini index [34] for pairwise class discrimination; this involved 6 possible pairwise comparisons (6 = 4(3)/2). The Gini index, *G*, is a measure of class impurity among objects assigned to a given node in a decision tree [34]. For a given tree node, 
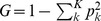
, where *p_k_* is the proportion of node members in class *k*, and *K* is the number of classes. Gini has range 0≤*G*≤1, and is equal to zero when there is class purity in the node, and equal to unity when *K*→∞ and all *p_k_* tend to zero. The first gene selected was therefore the best discriminating marker for the N and R classes, followed by the best discriminating marker for the N and L class pairs. Any markers that were the best for multiple pairs of classes were selected in the order of their first appearance among ranks. After filtering to identify marker order based on discrimination, k-nearest neighbor (KNN) classification analysis was performed with K = 5, so the KNN model was called 5NN. An odd value for K was chosen to prevent ties in the predicted class membership of nearest neighbors. Classification accuracy based on ten 10-fold cross-validation for predicting the correct true class label was performed using sets of the 1, 2,…, 9 ordered markers. Linear discriminant analysis and PCA were not performed because covariance (correlation) is undefined for one marker. Diagnostic screening was also determined for the 9-marker set to determine sensitivity, specificity, positive predictive value (PV+), and negative predictive value (PV−). Sensitivity is equal to the proportion of observations in a given class with the correct class prediction, whereas specificity is the proportion of observations not in a given class whose predicted membership is not in the given class. On the other hand, PV+ reflects the proportion of observations predicted to be in a given class that are truly in the class, while PV− is defined as the proportion of observations that are not predicted to be in a given class which are not in the given class.

## Results

### Radiation Response of DNA Repair Genes

**Figure 1:**
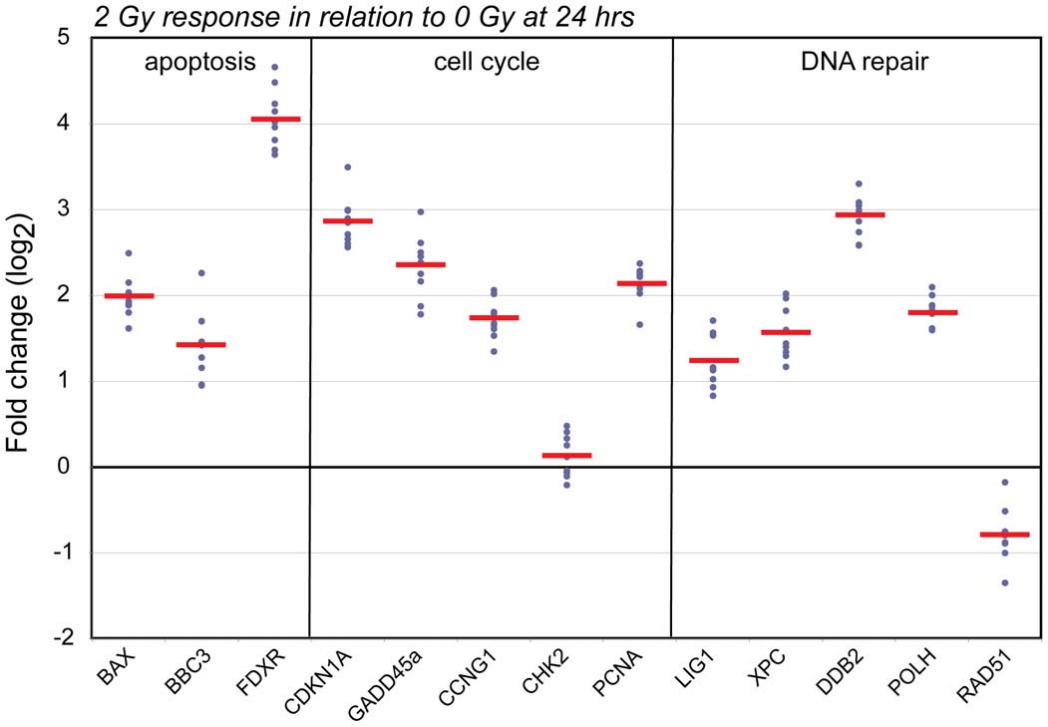
Radiation-induced transcriptional responses of DNA repair genes in the human *ex vivo* radiation blood model. Relative transcript level responses using human blood from 5 healthy human donors measured by quantitative RT-PCR analysis 24 hrs after 2 Gy exposure with respect to sham (0 Gy) transcript levels. Expression of the sham (0 Gy) and 2 Gy transcript responses were calculated relative to the average expression of *ACTB (β-Actin*). The delta Ct for *β-Actin* between sham and 2 Gy irradiated samples was <0.3 for all but one sample, which was excluded from this analysis. The fold-change for each gene between sham and irradiated samples was calculated using the delta-delta Ct method. Similar results were obtained when normalized using *GAPDH* expression as endogenous control (data not shown).

The radiation response of 40 genes associated with various aspects of DNA damage response (**Table S1**) was surveyed. Twelve genes (**Figure 1**) were significantly modulated in transcript response 24 hrs after *ex vivo* exposure to 2 Gy X-rays, relative to sham-irradiated samples (individual t-test 2.2E-06<p<0.03; **Figure S2**). Most of these genes (11 of 12) showed increased expression after exposure (**Figure 1**), ranging from 2.3-fold for *LIG1* to 17-fold for *FDXR*. *RAD51*, a key component of homologous recombination repair, was the only gene in this set that showed significant down regulation after exposure (2.5-fold). Expression responses for sham and irradiated samples showed little inter-individual variation and reproducible responses within donors sampled twice at approximately one month apart. These findings indicate that transcript responses of this panel of 12 DNA repair genes are robust biomarkers of radiation exposure in peripheral blood cells. Among these 12 genes, we found no overlap between sham and irradiated samples for 8 biomarkers (*BBC3, FDXR, CDKN1A, GADD45a, PCNA, XPC, DDB2* and *POLH*; individual T-test 2.24E-06<p<7.45E-04), and found only slight overlaps for the other 4 biomarkers (*BAX, CCNG1, LIG1* and *RAD51*; individual t-test 7.64E-04<p<2.57E-02) (**Figure S2**). CHK2 is included as an example of a gene that does not respond to radiation (**Figure 1**; p = 0.5). Our findings predict that when blood samples prior to exposure are not available, our panel of eight DNA repair markers can distinguish between 2 Gy irradiated and unirradiated individuals with 100% accuracy 24 hrs after a 2 Gy exposure.

### Validation of the Dose and Time Response Characteristics of Our 8-gene Transcript Panel in Independent Human *ex vivo* and *in vivo* Datasets

**Figure 2:**
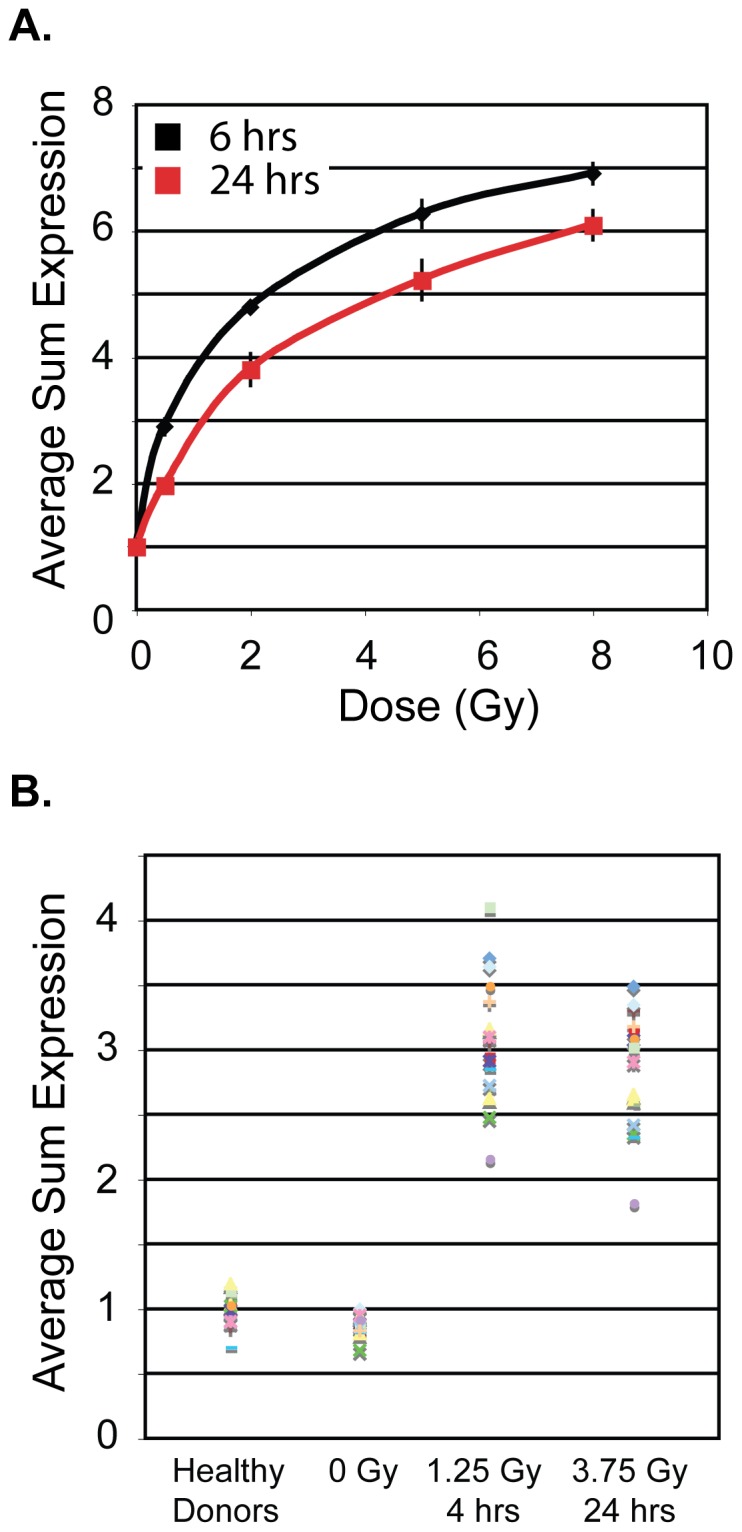
Independent human *ex vivo* and *in vivo* confirmation of the radiation response of the 8-gene panel. The robustness of our panel of 8 non-overlapping radiation biomarkers was confirmed using two published expression array data sets: (A) *ex vivo* irradiated (0, 0.5, 2, 5, 8 Gy) human blood samples obtained from five independent donors 6 and 24 hrs after radiation exposure (GSE8917; [10]) and (B) human *in vivo* irradiated blood samples obtained from patients undergoing total body irradiation (GSE20162; [12]). A. Shown is the average of the summed expression for the samples in each exposure group (+/− standard error) normalized to the average expression of the 0 Gy samples for each time-point. B. Shown is the plot of the summed expression of the 8-gene panel of each blood sample in the *in vivo* study, normalized to the average of the healthy donor samples.

We tested the dose response characteristics of our 8-gene panel (*BBC3, FDXR, CDKN1A, PCNA, XPC, GADD45a, DDB2* and *POLH*) using an independent public dataset containing microarray transcript expression data collected at 6 and 24 hrs after *ex vivo* exposures (sham, 0.5, 2, 5 and 8 Gy) in human blood from 5 independent donors. This analysis confirmed our primary finding of no overlap in transcript responses for any of the 8 genes between sham and irradiated samples at 24 hrs after 2 Gy exposures (**Figure S3**). We then investigated the dose and time response characteristics of our panel using the sum expression values of the 8 genes for each donor (**Figure 2A**). The average expression of our panel among the donor group was increased above sham for all dose groups tested at 6 hrs (p<5.5E-05) and 24 hrs (p<2.32E-04). The average sum expression at 6 hrs was consistently higher compared to 24 hrs for all doses tested (5.9E-04<p<0.02). Furthermore, the samples irradiated with 2 Gy were significantly different from those irradiated with 0.5 Gy (p<6.09E-03), 5 Gy (p<8.47E-03) or 8 Gy (p<1.65E-04) at 6 and 24 hrs, demonstrating the significant dose response characteristics of our panel.

We then tested whether our 8-gene panel could distinguish human patients receiving total body irradiation (TBI) from pre-irradiation patients and healthy controls. We obtained a public dataset containing microarray transcript expression data of blood collected from 14 independent healthy donors and from 18 patients who provided blood samples before TBI treatment, at 4 hrs after the first of three fractions of 1.25 Gy and at 20–24 hrs after the first fraction. We calculated the sum expression value for blood sample for each patient and control subject to investigate their variation across experimental groups. As shown in **Figure 2B**, there was no overlap in expression between TBI treated blood samples and pre-TBI and control group values. The average expression of the 8-gene panel after TBI treatment was increased above the levels of healthy control group and pre-TBI blood levels for both timepoints tested, at 4 hrs after first fraction (1.25 Gy; p<1.34E-11) and at 20–24 hrs after the first fraction (3.75 Gy; p<1.45E-11).

In a separate analysis of human *in vivo* radiation response, we compared our 8-gene panel against a 25-gene signature developed by Meadows et al [35] to distinguish healthy individuals and pre-irradiation patients from the irradiated patients. In their study, peripheral blood was obtained from TBI patients before irradiation and 6 hrs after 1.5–2.0 Gy. Peripheral blood was also obtained from a population of healthy control individuals. Interestingly, 5 of our eight biomarkers were present in their signature (*XPC*, *PCNA*, *CDKN1A*, *DDB2* and *BBC3*). These comparisons against two independent groups of blood samples from irradiated human patients provide compelling *in vivo* corroborative support for the utility of our 8-gene panel for radiation biodosimetry in blood cells.

### LPS Modulation of Transcript and Protein Expression in Irradiated Whole Blood Cultures

**Figure 3:**
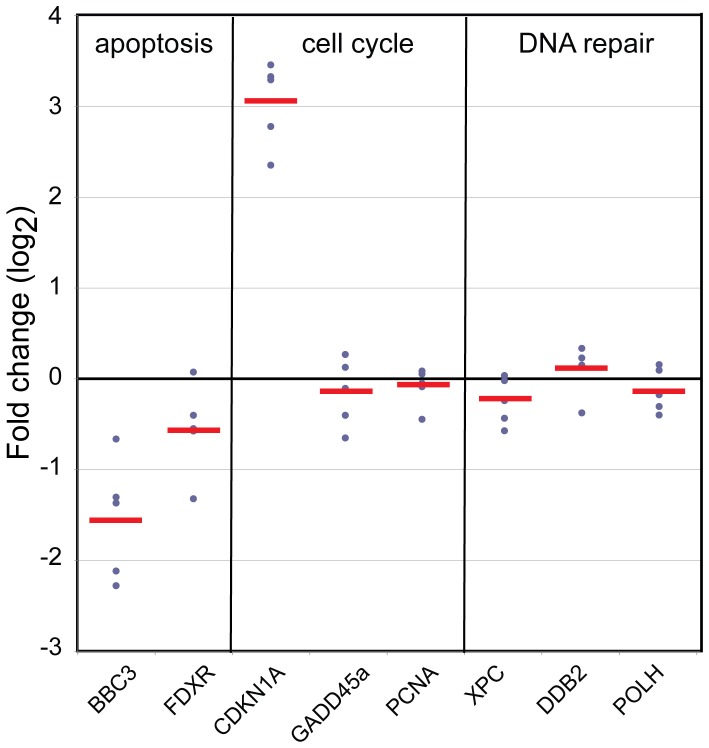
Effects of LPS treatment on radiation responsive DNA repair and cell cycle genes. Transcript level responses measured by quantitative RT-PCR analysis 24 hrs after LPS treatment of whole blood of two apoptosis, three cell cycle and three DNA repair genes with respect to transcript levels in untreated blood cultures. *CDKN1A* was strongly upregulated (∼8.2-fold) by LPS treatment alone in the absence of radiation exposure with little variation among donors. *BBC3* and *FDXR* expression was downregulated (∼3-fold and ∼1.5-fold, respectively) by LPS treatment. LPS treatment did not modulate expression levels of *GADD45a*, *PCNA*, *XPC*, *DDB2* and *POLH* (<1.5-fold change in expression compared to untreated samples). *ACTB* was used to normalize gene expression in samples in which the delta Ct of LPS treated vs untreated was less than 0.3 (donor 1.1, 3, 4.1, 5 and 5.1). *GAPDH* was not used to normalize since its levels varied depending on the presence of LPS (the average Ct difference between *GAPDH* in LPS treated and untreated samples was 0.54).

We investigated the specificity of the radiation response of our biomarkers in the *ex vivo* blood radiation model in the context of inflammatory stress simulated by LPS. We confirmed that LPS treatment (50 ng/ml) induced an inflammatory response in white blood cells by measuring the secretion of IL-6 and TNF-α into plasma of all donors tested, (**Figure S4**). LPS treatment showed no significant changes in baseline transcript expression in 5 biomarkers (fold-change <1.5; *GADD45a, PCNA, XPC, DDB2, POLH*) (**Figure 3**). Significant changes were observed in 3 biomarkers (**Figure 3**): >8-fold (±1.1) increase in *CDKN1A* and reduced expression in *BBC3* and *FDXR* (2.9-fold (±0.08) and 1.5-fold (±0.1), respectively).

**Figure 4:**
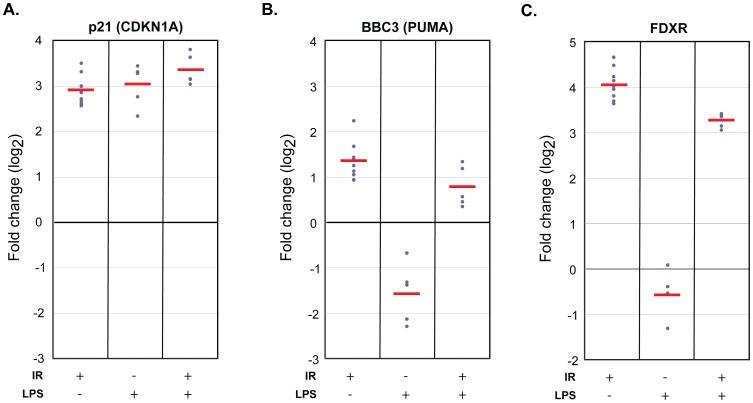
Radiation-induced transcript responses of *CDKN1A*, *BBC3* and *FDXR* are confounded by LPS treatment. Transcript level responses measured by quantitative RT-PCR analysis 24 hrs after exposure to 2 Gy, LPS treatment, and combined LPS and 2 Gy of whole blood of *CDKN1A* (A), *BBC3* (B), and *FDXR* (C) genes with respect to transcript levels in un-treated blood cultures. A. A radiation exposure of 2 Gy in the absence of LPS (left panel) or LPS treatment alone (middle panel) induced *CDKN1A* to approximately the same level at 24 hrs: 7.3 vs 8.2-fold, respectively (T-Test p = 0.47). LPS treatment in the presence of a 2 Gy radiation exposure induced *CDKN1A* expression ∼10.2-fold (right panel), which is a 1.4-fold increase compared to 2 Gy alone (T-test p = 0.03). B. In the absence of LPS, radiation induced *BBC3* ∼2.7-fold (left panel). LPS treatment alone (middle panel) suppresses *BBC3* ∼2.9-fold. LPS treatment in the presence of a 2 Gy radiation exposure induced *BBC3* expression ∼1.7-fold (right panel), a ∼1.6-fold decrease in *BBC3* expression when compared to 2 Gy alone (T-test p = 0.03). C. In the absence of LPS, radiation induced *FDXR* ∼17-fold (left panel). LPS treatment alone (middle panel) suppressed *FDXR* ∼1.5-fold. LPS treatment in the presence of a 2 Gy radiation exposure induced *FDXR* expression ∼10-fold (right panel), a ∼1.7-fold decrease in *FDXR* expression when compared to 2 Gy alone (T-test p = 1.2E-04).

**Figure 5:**
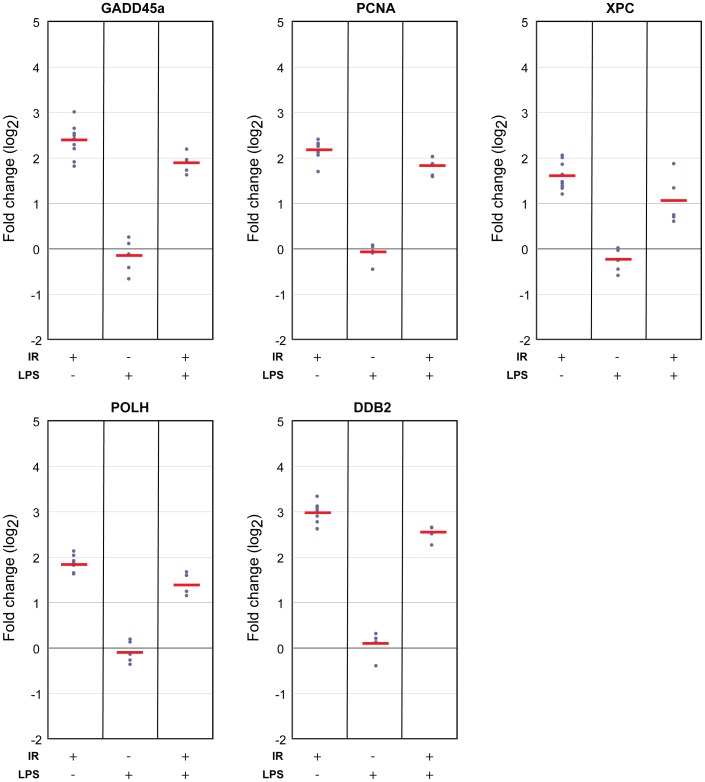
Radiation-induced transcript responses of *GADD45a*, *PCNA*, *XPC*, *POLH* and *DDB2* are minimally affected by LPS. Transcript level responses measured by quantitative RT-PCR analysis 24 hrs after exposure to 2 Gy, LPS treatment, and combined LPS and 2 Gy of whole blood of *GADD45a*, *PCNA*, *XPC*, *POLH* and *DDB2* genes with respect to transcript levels in un-treated blood cultures. Transcript levels of none of these five genes were significantly modulated 24 hrs after 2 Gy exposure in the presence of LPS compared to 2 Gy alone (fold-change <1.4-fold or p>0.03). Interestingly, the radiation response of all five genes was slightly suppressed by LPS treatment suggesting that LPS had a small effect on the 2 Gy response of these genes.

We then investigated the effect of LPS treatment on radiation response of the 8 genes in our panel, using blood cultures exposed to 2 Gy X-rays and co-treated with LPS (50 ng/ml). The strongest effect of LPS on the radiation responses was seen for *CDKN1A*, *BBC3* and *FDXR* (**Figure 4**; fold-change difference >1.4-fold and p<0.03). LPS modified the radiation response of *CDKN1A* by an additional 1.4-fold increase over the effects of radiation on *CDKN1A* expression alone (p = 0.03; **Figure 4A**). *BBC3* and *FDXR* expression, on the other hand, were repressed 1.6-fold (2.7-fold upregulated after radiation vs. 1.7-fold after radiation in the presence of LPS; p = 0.03) and 1.7-fold (17-fold upregulated after radiation vs. 10-fold after radiation in the presence of LPS; p = 1.2E-04) after radiation and subsequent culture in the presence of LPS in comparison to the effects of radiation alone (**Figure 4B and C**). The radiation responses of the remaining five biomarkers were not significantly altered by LPS treatment (**Figure 5**; <1.4-fold change or p>0.03).

**Figure 6:**
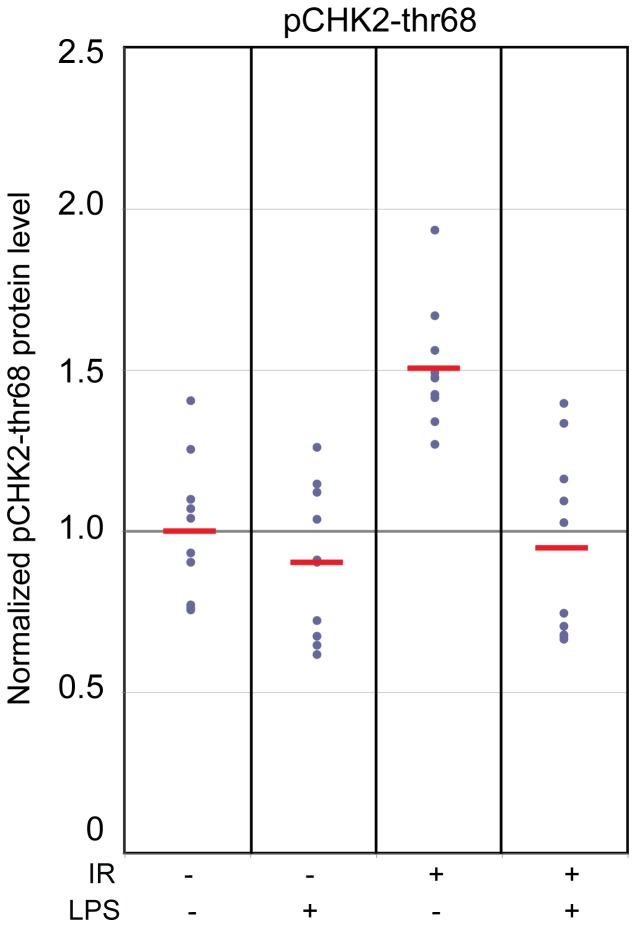
LPS mediated suppression of phosphorylated CHK2-thr68 protein at 24 hrs after 2 Gy exposures. Protein levels of phosphorylated CHK2-thr68 in protein lysate from cultured whole blood in the presence or absence of LPS (50 ng/ml) were measured by ELISA. Absorbance values were normalized with respect to the average pCHK2-thr68 level in unirradiated donors. In the absence of LPS, radiation induced CHK2-thr68 levels ∼1.6-fold (±0.1) relative to sham irradiated samples, whereas in the presence of LPS, CHK2-thr68 levels were indistinguishable from sham irradiated samples (p>0.4).

Our analyses of protein expression confirm that phosphorylated CHK2 protein is a radiation responsive biomarker [36], and demonstrate that the transcript levels of *CHK2* were unaffected by radiation only, LPS only, and co-exposure to both agents. Phosphorylated CHK2-thr68 protein levels showed a modest ∼1.6 (±0.1; p = 2.9E-05) fold increase in the whole blood *ex vivo* culture model at 24 hours post 2 Gy irradiation compared to sham (**Figure 6**). However, in the presence of LPS, this protein response was completely suppressed and indistinguishable from sham-irradiated samples without LPS co-treatment (p>0.4) (**Figure 6**). This is compelling evidence that pCHK2-thr68 may not be a suitable biomarker of radiation exposure when in the context of inflammatory stress. Interestingly, we found that by including pCHK2-thr68 as a member of the panel of biomarkers improved the discrimination of radiation-exposed individuals with inflammatory stress from those exposed to radiation alone. We arrived at this conclusion by comparing the relative protein radiation responses in our PBMC model compared to our data from the whole blood culture model. We measured pCHK2-thr68 as well as BAX protein levels in PBMCs of healthy donors at 6 and 24 hours after *ex vivo* exposure to 2 or 6 Gy X-rays. BAX was included in this study as a surrogate indicator for the role of apoptosis in these models (**Figure S5A)**. As expected, the pCHK2-thr68 responses were stronger at the earlier timepoint, while the BAX responses were stronger at later time (**Figure S5B)**. Both BAX and pCHK2-thr68 proteins showed significant increases at 6 and 24 hrs after both 2 and 6 Gy exposures (p<0.05) (**Figure S5C**). Due to the substantial variability in radiation response in the PBMC model system both among donors and between repeated blood draws of the same donor, we used the protein response data from the whole blood model in our further analyses.

### Multi-group Classification of Blood Samples by their Radiation and Inflammation Status

**Figure 7:**
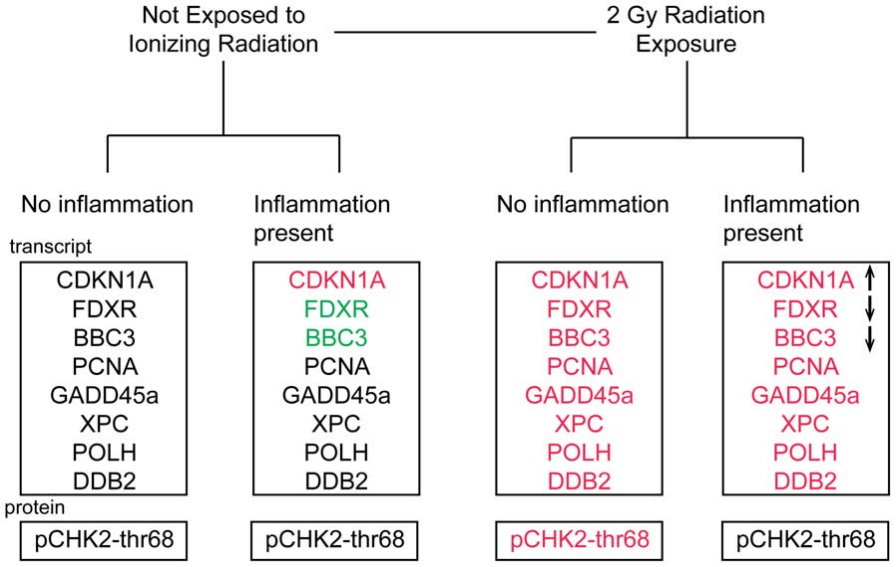
Transcript and protein panel discriminates 2 Gy exposure and unirradiated samples, independent of inflammation stress. In comparison to untreated sham samples, inflammation in the absence of radiation exposure upregulates *CDKN1A* (red) and downregulates *FDXR* and *BBC3* (green). Samples exposed to 2 Gy radiation only exhibit increased expression of all nine biomarkers, whereas subjects exposed to 2 Gy plus inflammation stress show modified induction of *CDKN1A*, *FDXR* and *BBC3* and abrogation of the phosphorylation of CHK2 protein. The arrows in the radiation and inflammation combined treatment group indicate the direction of expression relative to the radiation alone group.

**Figure 8:**
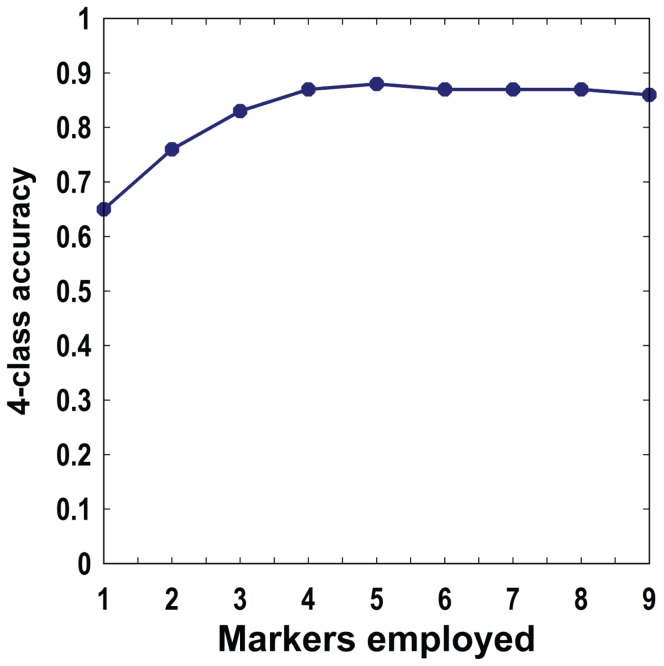
Classification (4-class) accuracy of the transcript and protein panel. Classification accuracy based on ten 10-fold cross-validation as a function of the number of markers considered, based on order determined during filtering with the Gini index. The four classes used in this analysis are: radiation only (R), inflammation stress only (L), combined exposures involving both radiation and LPS (RL), and samples with no radiation exposure and no LPS treatment (N). Marker order is: PCNA, CDKN1A, pCHK2-thr68, BBC3, FDXR, DDB2, XPC, POLH, and GADD45a. Maximum classification accuracy was 0.88 for the top 5-marker set.

We tested our combined nine-gene panel of eight transcript and one protein biomarkers in our *ex vivo* blood model to test its ability to discriminate among four exposure/treatment groups: radiation exposure only (R), inflammatory stress only (L), combined exposures with both radiation and LPS (RL), and samples with no radiation exposure or LPS treatment (N) (**Figure 7** **)**. Marker filtering with the Gini index (see methods) resulted in the following marker order: PCNA, CDKN1A, pCHK2-thr68, BBC3, FDXR, DDB2, XPC, POLH and GADD45a. **Figure 8** illustrates the cumulative 4-class accuracy for the sets of 1, 2,…,9 markers based on their order of selection during filtering. The overall accuracy for the single best marker, PCNA, was 0.65, and the accuracy of 0.88 was attained when 5 markers were used, after which the overall accuracy leveled off. **Table 1** lists the results of diagnostic screening analysis for the 9-marker set. The majority of sensitivity calculations approached 0.9 or greater. Specificity was in the 0.8–0.9 range. The positive predictive value (PV+) was unity (1.00) for the NL class, 0.96 for the L class, and below 0.8 for the R and RL classes, whereas the negative predictive value (PV−) ranged from 0.81–0.91. This analysis has identified a subpanel of 5 biomarkers that correctly assign individual blood samples to one of the four different experimental conditions with an overall classification accuracy of ∼ 90%.

**Table 1:** Classification sensitivity, specificity, predictive value positive (PV+), and predictive value negative (PV−) for the nine-gene panel.

Class	Sensitivity	Specificity	PV+	PV−
N	0.89	0.84	1	0.81
R	0.86	0.86	0.78	0.88
L	0.91	0.84	0.96	0.82
RL	0.76	0.89	0.69	0.91

Results are based on ten 10-fold cross-validation runs.

## Discussion

Our findings showcase the advantage of using blood cells and the expression of genes associated with the DNA repair for human radiation biodosimetry, and furthermore that these genes have the ability to discriminate between radiation dose and inflammation stress. In a survey of 40 DNA repair genes in the human peripheral blood cells *ex vivo* radiation model (**Table S1**), twelve genes showed more than two fold changes in transcript levels at 24 hours after 2 Gy exposures. These included the cell cycle regulators (*CDKN1A*, *GADD45a, PCNA* and *CCNG1*), apoptosis regulators (*BAX*, *BBC3* and *FDXR)* and genes involved in specific DNA repair functions (*XPC, DDB2, LIGI, POLH* and *RAD51*). We compared the responses to radiation and inflammation stress to develop a panel of 8 genes that we validated using publicly available expression datasets for (1) an independent group of donors in a blood *ex vivo* model, and (2) a second independent group of patients who provided blood samples before and after whole body radiation ([10], [12], [35], **Figure 2A**). Our findings support the strength of using DNA repair related genes to detect radiation exposure in the context of inflammation stress, which may become helpful for discriminating between worried-well, those exposed to medically significant doses of ionizing radiation and those experiencing inflammation stress (**Table 1**). By including protein expression markers we developed a 9-gene panel (8 transcripts and one protein marker) that correctly discriminated irradiated from unirradiated blood samples independent of the presence or absence of inflammation stress (**Figure 7**), with a ∼90% 4-group classification accuracy (**Figure 8**).

The DNA repair-associated genes we surveyed are regulated by TP53 signaling [37]. The TP53 tumor suppressor protein is central to cell signaling networks following cellular stressors, including DNA damage such as that caused by ionizing radiation. TP53 modulates the main DNA repair processes in eukaryotic cells (base excision repair (BER), nucleotide excision repair (NER), non-homologous end-joining (NHEJ) and homologous recombination (HR) along with direct roles in induction of DNA damage-induced cell cycle arrest and apoptosis. TP53 is activated after DNA damage through phosphorylation to function as a transcriptional regulator inducing expression of a number of downstream target genes that directly control cellular outcomes [38]. Activators of TP53 include CHK2, a serine/threonine kinase that, upon activation directly by ATM phosphorylation (e.g., threonine-68) or indirectly by other protein kinases (e.g., DNA-PKcs), acts as both a downstream signal transducer of DNA damage and an effector for DNA repair, checkpoint control and apoptosis [39]. In our study we did not observe changes in transcript expression of *CHK2* following irradiation, consistent with the role of CHK2 as an upstream mediator of TP53 rather than a downstream target, however, an increase in phosphorylated CHK2 protein was observed. Phosphorylation of TP53 at serine-20 by CHK2 prevents MDM2-mediated TP53 degradation. This enhancement of TP53 stability allows for the continuance of downstream DNA damage response pathways including apoptosis, of which BAX is an effector [40]. CHK2, a direct substrate of ATM, is an earlier DNA damage response protein than BAX. Hirao et al. [41] observed by Western blot that CHK2 levels in both sham- and 5 Gy irradiated wild-type mouse thymocytes precede BAX up until 6 h post-irradiation, which is consistent with our protein ELISA results post-irradiation.

Our radiation-response results in the *ex vivo* blood model are consistent with previous human studies [6], [8], [10], [23]–[25], [42] with the exception of *RAD51*, which showed a decrease in expression in our study [43]. A recent study in mice of radiation effects on gene expression showed significant increases in expression of *CDKN1A, BBC3* and *GADD45a* at 24 hrs after 2 Gy whole body irradiation [42]. However, in that study *DDB2* was downregulated and no significant changes were observed for *FDXR* or *XPC,* which is inconsistent with our results and those of others in humans irradiated *ex vivo* [10]. Expression of *GADD45a, LIG1* and *XPC* were decreased at 24 hours after 6 Gy IR in mice, whereas we observed increased expression at 24 hrs after 2 Gy in our *ex vivo* human blood culture model consistent with published human *ex vivo* and *in vivo* literature [7], [12], [30]. Also, our use of a 2 Gy exposure (rather than 6 Gy used in a prior mouse study [30]) is more relevant for radiation biodosimetry because individuals having a radiation exposure dosage of less than 2 Gy require no immediate treatment as opposed to those having a dosage higher than 2 Gy. The inherent differences between murine and human assays emphasize the importance of using human model systems to validate biomarkers for human radiation biodosimetry. Our study investigates the blood of unrelated people and we confirm our findings in a separate independent group of unrelated people, suggesting that interindividual variation in the transcript response is not a major factor for the genes in our panel.

Understanding the effects of confounding factors, such as inflammation stress, on radiation-responsive biomarkers is important for assessing their utility in radiation biodosimetry in practical human exposure scenarios [1], [2], . Of the 8 radiation-responsive genes in our study, only three (*CDKN1A, FDXR* and *BBC3*) were confounded by LPS-induced inflammation stress. *CDKN1A* is a canonical marker of DNA damage response and has been proposed as a biomarker of radiation exposure [7], [42]. While cigarette smoking did not confound the radiation response of *CDKN1A* [29], our study shows that inflammatory stress induced *CDKN1A* transcript levels in the absence of radiation exposure. Our finding seriously undermines the promise of *CDKN1A* as a predictive tool for radiation exposure in individuals suffering simultaneous inflammatory stress. Studies in the murine central nervous system also identified *CDKN1A* as an inflammatory response gene [44] and LPS exposure upregulated *CDKN1A* transcripts in mice [30]. LPS-induced and the radiation-induced *CDKN1A* responses were indistinguishable in our human blood model, while in the mouse the upregulation of *CDKN1A* at 24 hours after LPS injection did not mask the ability to detect a radiation response [30]. This difference in murine vs human responses might be attributed to the differences in LPS dosage (50 ng/ml in our study vs. 0.3 mg/kg which equals 7.2 µg per mouse), LPS bioavailability and species differences in response.

We have made the new observation that LPS co-treatment confounds the transcript response of *FDXR* and *BBC3,* also compromising their utility as radiation biodosimeters. The pro-apoptotic gene, *BBC3*, is responsible for induction of apoptosis pathways following DNA damage. Whole blood cultured in the presence of LPS repressed the expression of *BBC3* ∼2.5-fold. Co-treatment with LPS and radiation diminished *BBC3* transcripts compared to either LPS alone or radiation alone. Consistent with our finding, LPS suppressed apoptosis in human blood monocytes [45], but some studies found opposite responses [33]. The transcription of *BBC3* is regulated by a complex combination of pro-apoptotic and pro-survival mechanisms [46], suggesting that LPS may suppress *BBC3* transcription in blood cultures through activation of pro-survival signals. In contrast to our findings, Tucker and colleagues observed a marginal confounding effect of LPS treatment on the radiation response of *BBC3* in mice [30], again emphasizing the importance of validating biomarker panels in a human model.

The increases in protein levels of phosphorylated CHK2 after radiation-alone exposures were fully suppressed in the presence of LPS, also undermining it as a useful protein biomarker for radiation response in the context of inflammation stress. CHK2 protein is phosphorylated in response to DNA damage which activates the protein [13], [36]. While we demonstrate that LPS co-treatment fully abrogates this radiation-induced CHK2 phosphorylation process, the underlying mechanisms for this confounding effect remain unclear.

The LPS-modified *CDKN1A, FDXR* and *BBC3* transcript levels were remarkably uniform among donors, even though the secretions of IL-6 and TNF-α two genes well-known to be induced by LPS, were more variable (**Figure S4**). The levels of LPS-induced IL-6 and TNF-α were highly correlated (R^2^ = 0.8; **Figure S6**). Among 4 of the donors, IL-6 and TNF-α levels in the first blood draw were nearly identical to those in the second blood draw, 1 month later. These findings point to the hypothesis that the induction of inflammatory response genes IL-6 and TNF-α depend on genetic background, while the inductions of *CDKN1A* and *BBC3* are more ‘switch-like’. This would predict that other confounding stimuli might also affect *CDKN1A* and *BBC3* expression.

Our research has identified a small panel of DNA repair-related biomarkers that distinguish among human blood samples from four radiation exposure scenarios: no radiation exposure, 2 Gy radiation exposure only, inflammation stress without radiation exposure, and combined 2 Gy exposure plus inflammation stress. Independent validation for dose and time response and with *in vivo* total body irradiated samples further supports the utility of these biomarkers for clinical applications, accident scenarios and other situations involving potential radiation exposure. Future studies will be needed to evaluate our panel for effects of gender, age, and inter-individual variations, to examine the influence of differential radiation cytotoxicities of the white cell subtypes on expression biodosimetry [47], and to investigate the radiation specificity of our panel using other inflammation, chemical, and physical stressors that are relevant for human radiation biodosimetry applications in various hypothetical exposure scenarios.

## Supporting Information

Figure S1 **Standard curves for ELISAs.** BAX, IL-6 and TNF-α representative standard curves are shown. pCHK2-thr68 did not use a standard curve.
(PDF)

Figure S2 **Transcript level radiation responses of twelve DNA repair-related biomarkers.** Relative expression of the sham (0 Gy) and 2 Gy transcript responses were calculated relative to the mean expression of *ACTB* (*β-Actin*). Each symbol represents mean of 3 replicate relative expression levels for the designated DNA repair genes from a blood collection of a single donor. Data are plotted for 5 donors, each donating two blood samples. The delta Ct for *β-Actin* between sham and 2 Gy irradiated samples was <0.3 for all but one sample, which was excluded from this analysis. A two-sided T-test was performed on the distribution of expression levels between sham and irradiated samples (p-values are shown in the lower right of each box-plot).
(PDF)

Figure S3 **Transcript level radiation responses of eight DNA repair-related biomarkers in an independent dataset.** Normalized expression intensities of the sham (0 Gy) and 2 Gy transcript responses are shown. Each symbol represents expression levels for the designated DNA repair gene from a blood collection of a single donor. Data are plotted for 5 donors. A two-sided T-test was performed on the distribution of expression levels between sham and irradiated samples (p-values are shown in the lower right of each box-plot).
(PDF)

Figure S4 **Increased plasma protein levels of IL-6 and TNF-**α **in LPS-treated human blood in 24-hour culture.** Levels of IL-6 (A) and TNF-α (B) were measured by ELISA in human whole blood culture after LPS treatment (50 ng/ml) for 24 hrs. Blood cultures, from five unique donors, each sampled twice (second samples from each donor indicated by x.1), were assessed for IL-6 and TNF-α protein levels in plasma at 24 hrs by ELISA. Concentrations of protein (mg/ml) in each sample and treatment group are listed in the tables below the figures. In the absence of LPS-induced inflammatory stress, there is little detectable protein for either IL-6 or TNF-α. The presence of LPS induces up to a 7000-fold increase in protein levels for IL-6 (donor 4) and a 3000-fold increase in TNF-α (donor 4). Responses varied across donors but were consistent between the two different protein markers of inflammatory stress for each donor (donor 3 and donor 4 showed the greatest induction of both proteins).
(PDF)

Figure S5 **Radiation-induced increased protein levels of BAX and phosphorylated CHK2-thr68 in human ex vivo quiescent PBMC.** A. BAX and pCHK2-thr68 responses by ELISA after 0, 2 or 6 Gy at 6 and 24 hrs in independent replicate culture flasks from the same blood sample of two donors produce minimal technical variability (R^2^ = 0.95 for pCHK2-thr68; R^2^ = 0.92 for BAX). B. Levels of BAX and pCHK2-thr68 were measured by ELISA in unstimulated PBMC after 0, 2, or 6 Gy ionizing radiation. PBMC cultures from six or five unique donors were assessed for BAX and pCHK2-thr68 protein levels at 6 hrs and 24 hrs by ELISA. Data were normalized with respect to sham for each timepoint. Repeat draws from the same donor ∼ 1 month after the first blood draw are indicated with a “.1” after the donor identifier. C. T-test results (t-statistics and p-values in parentheses) identify significant mean differences in BAX and pCHK2-thr68 ELISA data for the 0- vs. 2 Gy and 0- vs. 6 Gy groups at 6 hrs and 24 hrs after irradiation.
(PDF)

Figure S6 **Correlation between IL-6 and TNF-**α **responses in LPS treated whole blood cultures.** Secretion of IL-6 and TNF-α were measured by ELISA for 5 donors. Each donor is represented with a different color. All donors were sampled twice at least one month apart. Note that the TNF-α and IL-6 secretory response after LPS treatment is variable among donors, but highly correlated between the replicate blood draws for each donor with the exception of the donor represented in red.
(PDF)

Table S1 **Target genes selected from DNA damage response pathways for transcript analysis.**
(PDF)

Table S2 **Average absorbance ranges of ELISA measurements.**
(PDF)
