# Supplementary figures and images for: DNA Repair and Cell Cycle Biomarkers of Radiation Exposure and Inflammation Stress in Human Blood
Source: PLoS One. 2012 Nov 7;7(11):e48619. doi: 10.1371/journal.pone.0048619 (PMC3492462; doi:10.1371/journal.pone.0048619)

Figure S1. Standard curves for ELISAs.

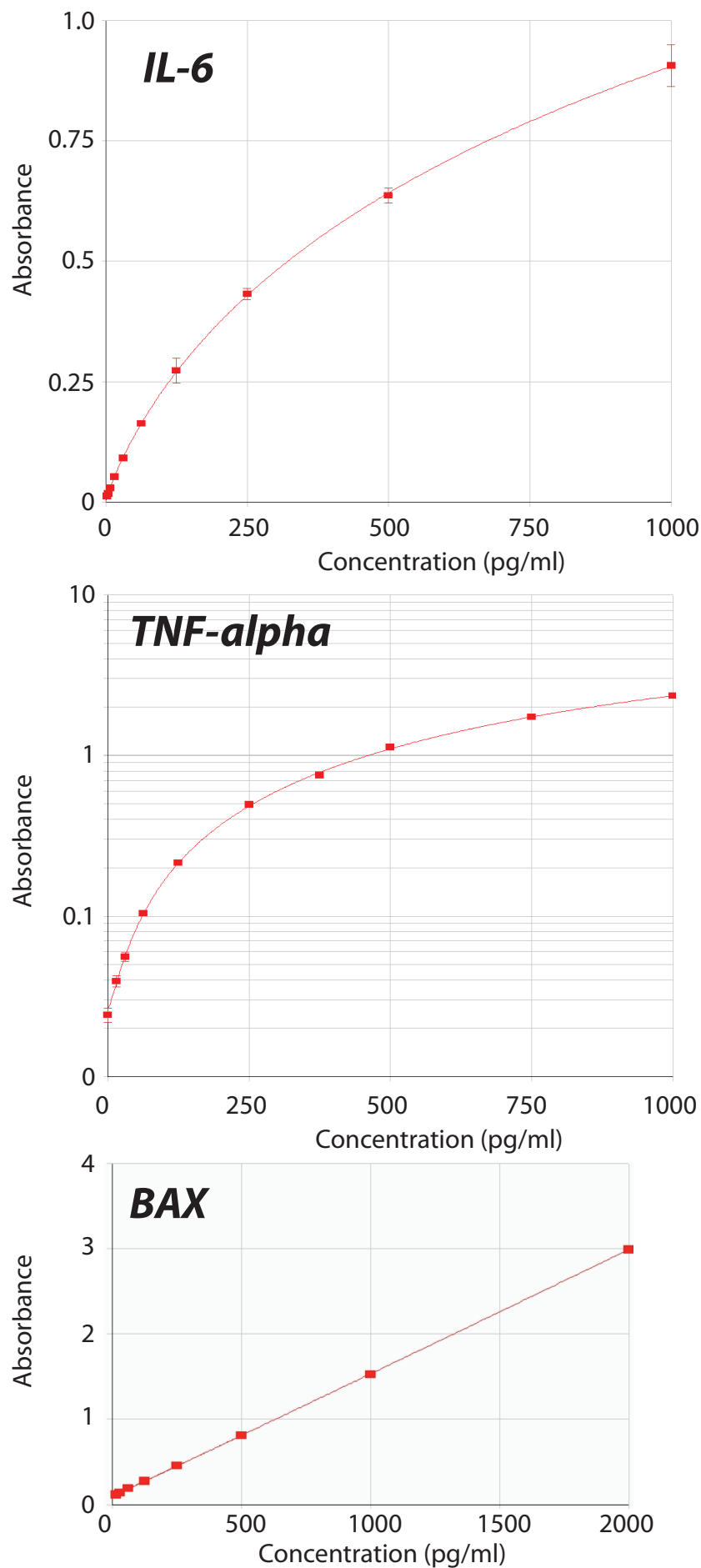

Supplement: Figure S1 — Standard curves for ELISAs. BAX, IL-6 and TNF-α representative standard curves are shown. pCHK2-thr68 did not use a standard curve. (PDF) [file pone.0048619.s001.pdf]

Figure S2. Transcript level radiation responses of twelve DNA-repair-related biomarkers.

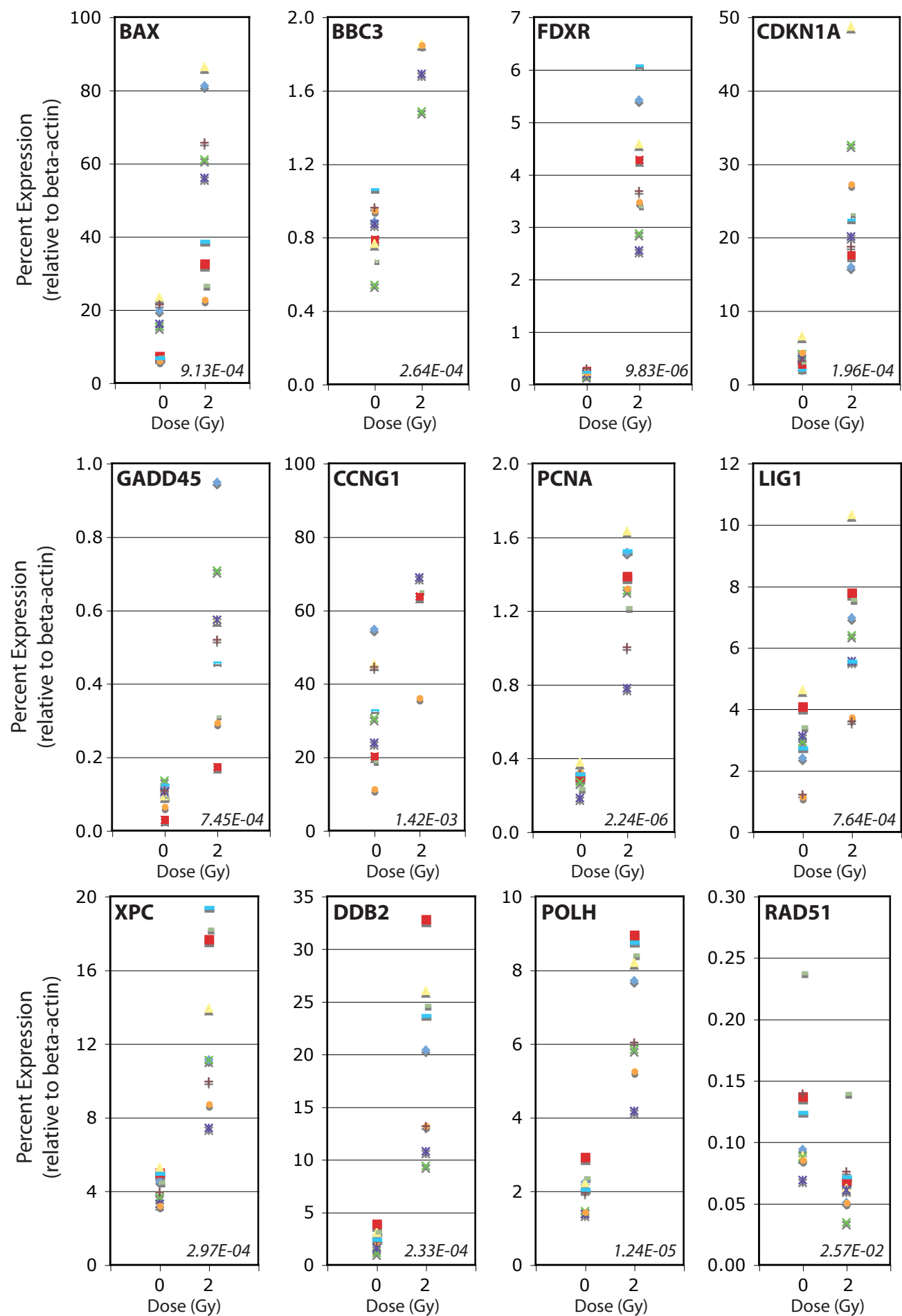

Supplement: Figure S2 — Transcript level radiation responses of twelve DNA repair-related biomarkers. Relative expression of the sham (0 Gy) and 2 Gy transcript responses were calculated relative to the mean expression of ACTB (β-Actin). Each symbol represents mean of 3 replicate relative expression levels for the designated DNA repair genes from a blood collection of a single donor. Data are plotted for 5 donors, each donating two blood samples. The delta Ct for β-Actin between sham and 2 Gy irradiated samples was <0.3 for all but one sample, which was excluded from this analysis. A two-sided T-test was performed on the distribution of expression levels between sham and irradiated samples (p-values are shown in the lower right of each box-plot). (PDF) [file pone.0048619.s002.pdf]

Figure S6. Correlation between IL-6 and TNF-alpha responses in LPS treated whole blood cultures.

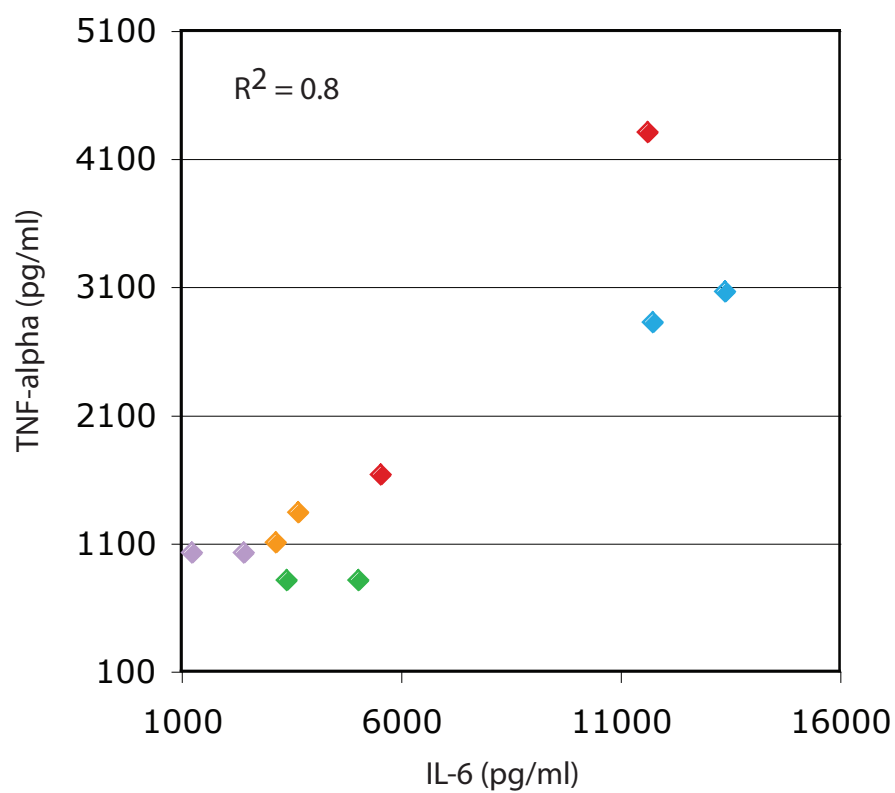

Supplement: Figure S6 — Correlation between IL-6 and TNF-α responses in LPS treated whole blood cultures. Secretion of IL-6 and TNF-α were measured by ELISA for 5 donors. Each donor is represented with a different color. All donors were sampled twice at least one month apart. Note that the TNF-α and IL-6 secretory response after LPS treatment is variable among donors, but highly correlated between the replicate blood draws for each donor with the exception of the donor represented in red. (PDF) [file pone.0048619.s006.pdf]
