# Supplementary material for: DNA Repair and Cell Cycle Biomarkers of Radiation Exposure and Inflammation Stress in Human Blood
Source: PLoS One. 2012 Nov 7;7(11):e48619. doi: 10.1371/journal.pone.0048619 (PMC3492462; doi:10.1371/journal.pone.0048619)

Figure S3. Transcript level radiation responses of eight DNA repair related biomarkers in an independent dataset.

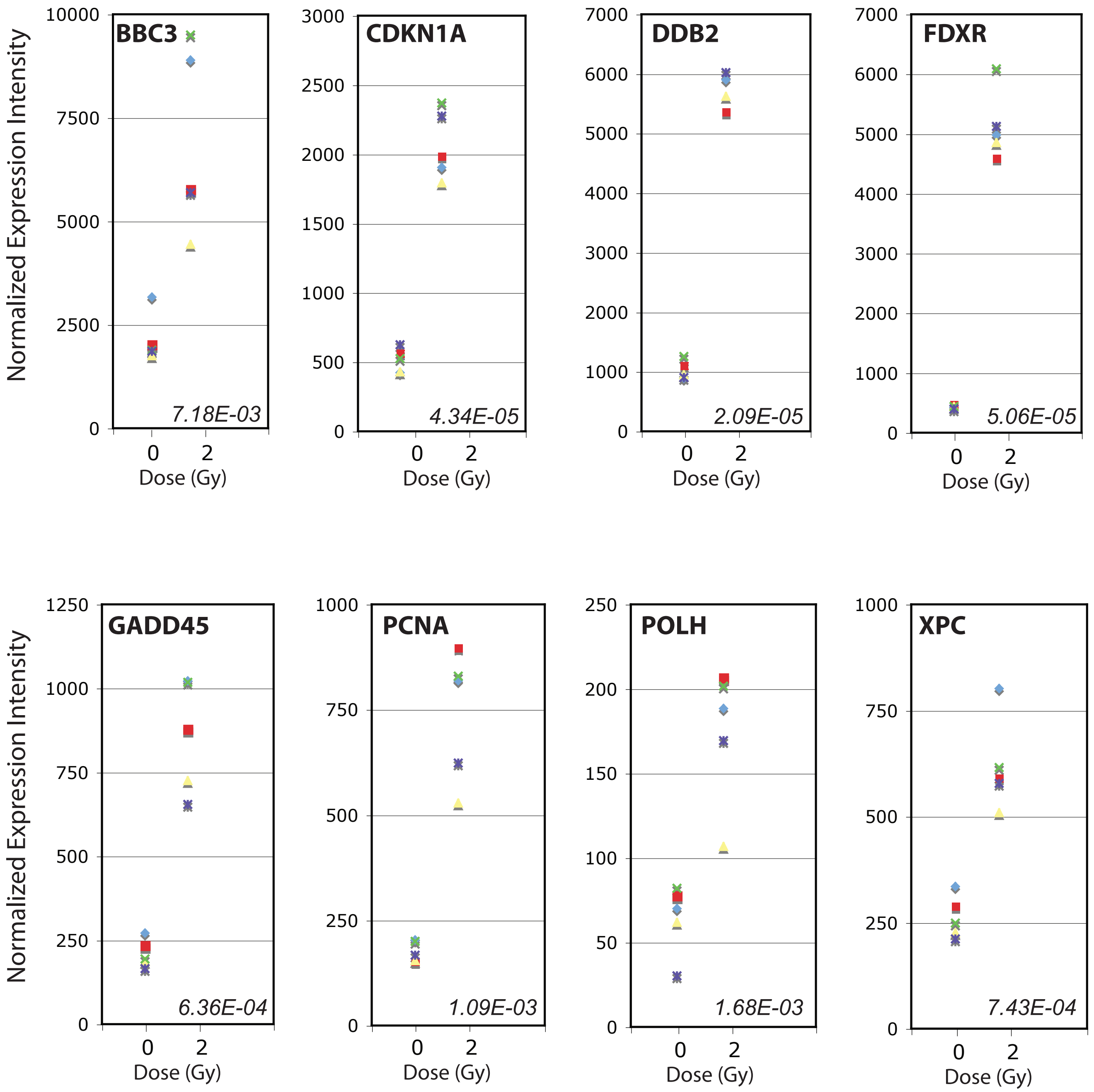

Supplement: Figure S3 — Transcript level radiation responses of eight DNA repair-related biomarkers in an independent dataset. Normalized expression intensities of the sham (0 Gy) and 2 Gy transcript responses are shown. Each symbol represents expression levels for the designated DNA repair gene from a blood collection of a single donor. Data are plotted for 5 donors. A two-sided T-test was performed on the distribution of expression levels between sham and irradiated samples (p-values are shown in the lower right of each box-plot). (PDF) [file pone.0048619.s003.pdf]
