# Supplementary material for: DNA Repair and Cell Cycle Biomarkers of Radiation Exposure and Inflammation Stress in Human Blood
Source: PLoS One. 2012 Nov 7;7(11):e48619. doi: 10.1371/journal.pone.0048619 (PMC3492462; doi:10.1371/journal.pone.0048619)
Supplement: Figure S4 — Increased plasma protein levels of IL-6 and TNF-α in LPS-treated human blood in 24-hour culture. Levels of IL-6 (A) and TNF-α (B) were measured by ELISA in human whole blood culture after LPS treatment (50 ng/ml) for 24 hrs. Blood cultures, from five unique donors, each sampled twice (second samples from each donor indicated by x.1), were assessed for IL-6 and TNF-α protein levels in plasma at 24 hrs by ELISA. Concentrations of protein (mg/ml) in each sample and treatment group are listed in the tables below the figures. In the absence of LPS-induced inflammatory stress, there is little detectable protein for either IL-6 or TNF-α. The presence of LPS induces up to a 7000-fold increase in protein levels for IL-6 (donor 4) and a 3000-fold increase in TNF-α (donor 4). Responses varied across donors but were consistent between the two different protein markers of inflammatory stress for each donor (donor 3 and donor 4 showed the greatest induction of both proteins). (PDF) [file pone.0048619.s004.pdf]

Figure S4. Increased plasma protein levels of IL-6 and TNF-α in LPS-treated human blood in 24-hour culture.

A.

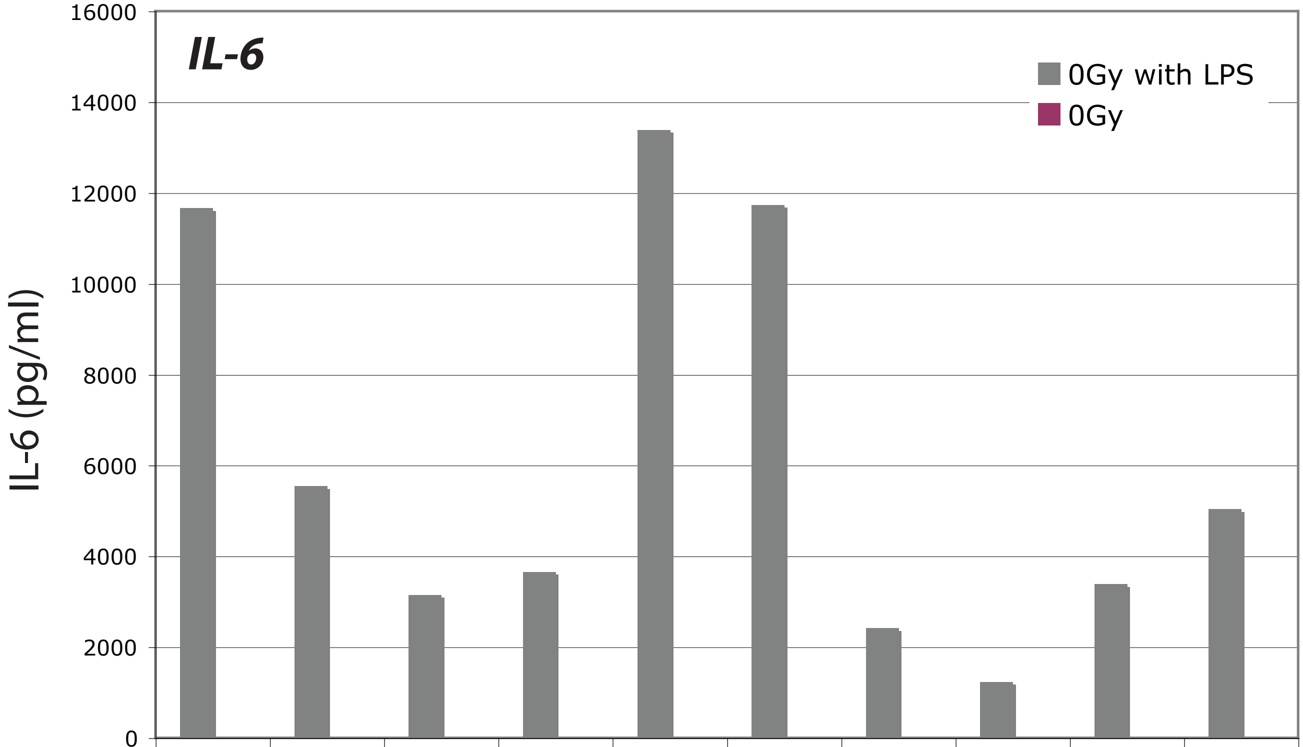

Donor	3	3.1	1	1.1	4	4.1	2	2.1	5	5.1
0Gy with LPS	11672	5550.1	3153	3664.7	13393	11745	2424.8	1242.6	3399	5043.8
0Gy	1.5087	1.5693	1.4364	1.3289	2.1543	1.7779	1.2697	1.0252	1.5572	3.7754

B.

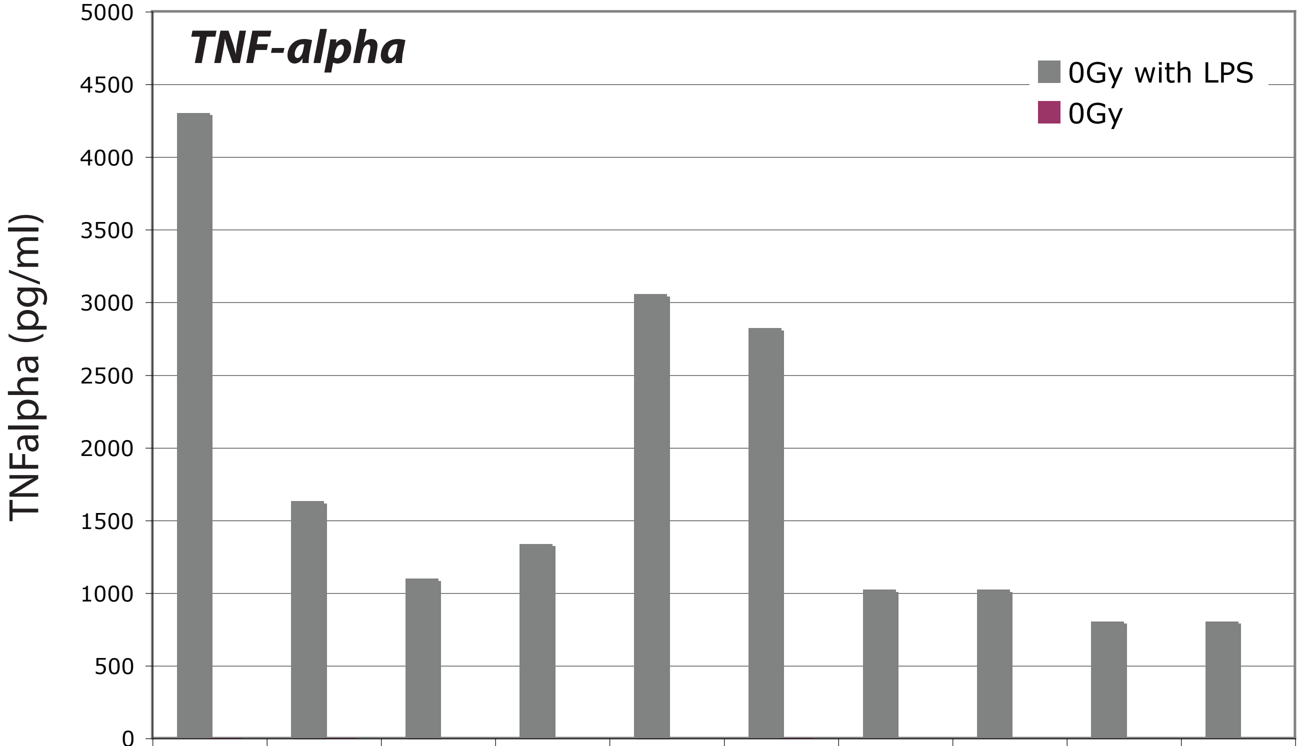

Donor	3	3.1	1	1.1	4	4.1	2	2.1	5	5.1
0Gy with LPS	4307	1635.2	1102.6	1340.5	3059.6	2824.6	1025.9	1025	808.85	807.89
0Gy	4.2542	7.3866	0	0	0	7.2623	0	0	0	1.7498
