# Supplementary material for: DNA Repair and Cell Cycle Biomarkers of Radiation Exposure and Inflammation Stress in Human Blood
Source: PLoS One. 2012 Nov 7;7(11):e48619. doi: 10.1371/journal.pone.0048619 (PMC3492462; doi:10.1371/journal.pone.0048619)
Supplement: Figure S5 — Radiation-induced increased protein levels of BAX and phosphorylated CHK2-thr68 in human ex vivo quiescent PBMC. A. BAX and pCHK2-thr68 responses by ELISA after 0, 2 or 6 Gy at 6 and 24 hrs in independent replicate culture flasks from the same blood sample of two donors produce minimal technical variability (R2 = 0.95 for pCHK2-thr68; R2 = 0.92 for BAX). B. Levels of BAX and pCHK2-thr68 were measured by ELISA in unstimulated PBMC after 0, 2, or 6 Gy ionizing radiation. PBMC cultures from six or five unique donors were assessed for BAX and pCHK2-thr68 protein levels at 6 hrs and 24 hrs by ELISA. Data were normalized with respect to sham for each timepoint. Repeat draws from the same donor ∼ 1 month after the first blood draw are indicated with a “.1” after the donor identifier. C. T-test results (t-statistics and p-values in parentheses) identify significant mean differences in BAX and pCHK2-thr68 ELISA data for the 0- vs. 2 Gy and 0- vs. 6 Gy groups at 6 hrs and 24 hrs after irradiation. (PDF) [file pone.0048619.s005.pdf]

Figure S5. Radiation-induced increased protein levels of BAX and phosphorylated CHK2-thr68 in human ex vivo quiescent PBMC.

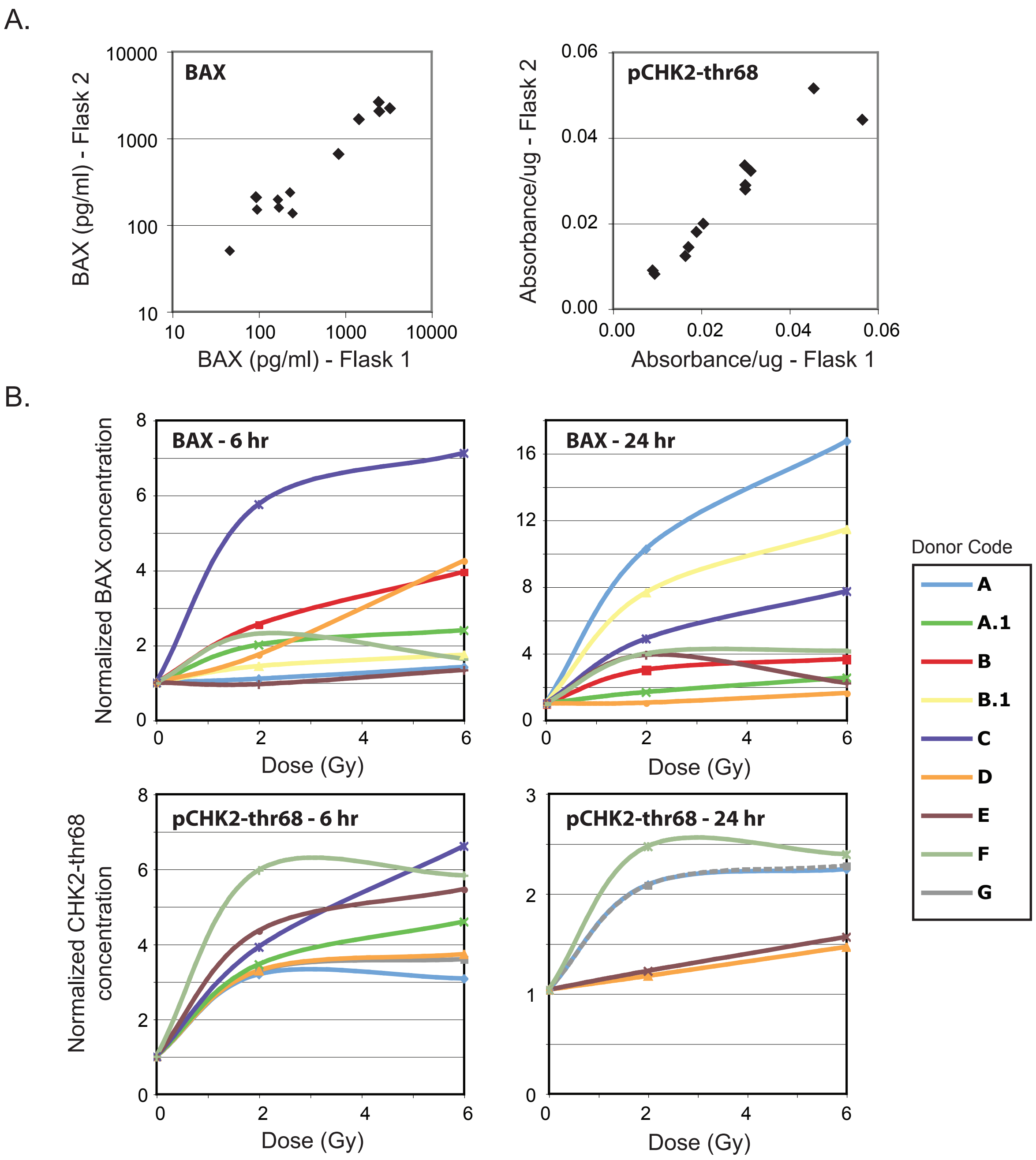

C.

t-statistic (p-value)	BAX 6 hr	BAX 24hr	pCHK2-thr68 6 hr	pCHK2-thr68 24 hr
**2 Gy vs sham**	-2.09 (0.046)	-4.95 (<0.0001)	-9.23 (<0.0001)	-2.54 (0.02)
**6 Gy vs sham**	-3.14 (0.004)	-5.41 (<0.0001)	-10.47 (<0.0001)	-3.33 (0.004)
