# Supplementary material for: DNA Repair and Cell Cycle Biomarkers of Radiation Exposure and Inflammation Stress in Human Blood
Source: PLoS One. 2012 Nov 7;7(11):e48619. doi: 10.1371/journal.pone.0048619 (PMC3492462; doi:10.1371/journal.pone.0048619)
Supplement: Table S1 — Target genes selected from DNA damage response pathways for transcript analysis. (PDF) [file pone.0048619.s007.pdf]

Table S1. Target genes selected from DNA damage response pathways for transcript analysis.

| Gene Symbol | Alternative Symbol | Entrez Gene Name | Process* | AOD** |
|---|---|---|---|---|
| APEX1 | APE1 | APEX nuclease (multifunctional DNA repair enzyme) 1 | BER | Hs00172396_m1 |
| BAX | | BCL2-associated X protein | apoptosis | Hs00180269_m1 |
| BBC3 | | BCL2 binding component 3 | apoptosis | Hs00248075_m1 |
| CCNG1 | | cyclin G1 | cell cycle | Hs00171112_m1 |
| CDKN1A | p21 | cyclin-dependent kinase inhibitor 1A (p21, Cip1) | cell cycle | Hs00355782_m1 |
| CHEK2 | CHK2 | CHK2 checkpoint homolog (S. pombe) | cell cycle | Hs00200485_m1 |
| DDB2 | | damage-specific DNA binding protein 2, 48kDa | NER | Hs03044953_m1 |
| ERCC1 | | excision repair cross-complementing rodent repair deficiency, complementation group 1 | NER | Hs01012158_m1 |
| ERCC2 | XPD | excision repair cross-complementing rodent repair deficiency, complementation group 2 | NER | Hs00361161_m1 |
| ERCC3 | XPB | excision repair cross-complementing rodent repair deficiency, complementation group 3 | NER | Hs01554450_m1 |
| ERCC4 | XPF | excision repair cross-complementing rodent repair deficiency, complementation group 4 | NER | Hs01063538_m1 |
| ERCC5 | XPG | excision repair cross-complementing rodent repair deficiency, complementation group 5 | NER | Hs01557031_m1 |
| ERCC6 | CSB | excision repair cross-complementing rodent repair deficiency, complementation group 6 | NER | Hs00972920_m1 |
| FDXR | | ferredoxin reductase | mitochondrial electron transport | Hs01031624_m1 |
| FEN1 | | flap structure-specific endonuclease 1 | BER | Hs00748727_s1 |
| GADD45A | | growth arrest and DNA-damage-inducible, alpha | cell cycle | Hs99999173_m1 |
| LIG1 | | ligase I, DNA, ATP-dependent | BER | Hs01553527_m1 |
| LIG3 | | ligase III, DNA, ATP-dependent | BER | Hs00242692_m1 |
| MLH1 | | mutL homolog 1, colon cancer, nonpolyposis type 2 (E. coli) | MMR | Hs00179866_m1 |
| MSH2 | | mutS homolog 2, colon cancer, nonpolyposis type 1 (E. coli) | MMR | Hs00953523_m1 |
| MSH3 | | mutS homolog 3 (E. coli) | MMR | Hs00989003_m1 |
| MSH6 | | mutS homolog 6 (E. coli) | MMR | Hs00264721_m1 |
| NTHL1 | NTH1 | nth endonuclease III-like 1 (E. coli) | BER | Hs00959764_m1 |
| OGG1 | | 8-oxoguanine DNA glycosylase | BER | Hs00213454_m1 |
| PARP1 | | poly (ADP-ribose) polymerase 1 | BER | Hs00242302_m1 |
| PARP3 | | poly (ADP-ribose) polymerase family, member 3 | DSB repair | Hs00193946_m1 |
| PCNA | | proliferating cell nuclear antigen | BER | Hs00696862_m1 |
| PMS1 | | PMS1 postmeiotic segregation increased 1 (S. cerevisiae) | MMR | Hs00922262_m1 |
| POLB | | polymerase (DNA directed), beta | BER | Hs01099715_m1 |
| POLH | | polymerase (DNA directed), eta | BER | Hs00982625_m1 |
| POLI | | polymerase (DNA directed) iota | Other | Hs00200488_m1 |
| POLK | | polymerase (DNA directed) kappa | Other | Hs00211963_m1 |
| RAD51 | | RAD51 homolog (S. cerevisiae) | DSB repair | Hs00153418_m1 |
| REV1 | | REV1 homolog (S. cerevisiae) | Other | Hs00249411_m1 |
| RFC1 | RFC | replication factor C (activator 1) 1, 145kDa | NER | Hs00161340_m1 |
| RPAIN | RIP | RPA interacting protein | NER | Hs00260434_m1 |
| XPA | | xeroderma pigmentosum, complementation group A | NER | Hs00166045_m1 |
| XPC | | xeroderma pigmentosum, complementation group C | NER | Hs01104206_m1 |
| XRCC1 | | X-ray repair complementing defective repair in Chinese hamster cells 1 | BER | Hs00959834_m1 |
| XRCC6 | KU70 | X-ray repair complementing defective repair in Chinese hamster cells 6 | DSB repair | Hs00995282_g1 |

*BER: base excision repair; NER: nucleotide excision repair; MMR: mismatch repair; DSB repair: double strand break repair

**AOD: Assay On Demand. Applied Biosystems TaqMan identification
