# Supplementary material for: DNA Repair and Cell Cycle Biomarkers of Radiation Exposure and Inflammation Stress in Human Blood
Source: PLoS One. 2012 Nov 7;7(11):e48619. doi: 10.1371/journal.pone.0048619 (PMC3492462; doi:10.1371/journal.pone.0048619)
Supplement: Table S2 — Average absorbance ranges of ELISA measurements. (PDF) [file pone.0048619.s008.pdf]

Table S2. Average absorbance ranges of ELISA measurements.

Average absorbance levels normalized to the reference absorbance value for BAX and pCHK2-thr68 in protein lysates of ex vivo irradiated PBMC.

Dose	BAX		pCHK2-thr68	
Time	6 hr	24 hr	6 hr	24 hr
0 Gy	0.17 (0.11 - 0.34)	0.23 (0.08 - 0.67)	0.17 (0.12 - 0.23)	0.17 (0.12 - 0.26)
2 Gy	0.24 (0.12 - 0.59)	0.36 (0.20 - 0.87)	0.63 (0.37 - 0.80)	0.32 (0.15 - 0.47)
6 Gy	0.28 (0.15 - 0.67)	0.47 (0.15 - 1.31)	0.71 (0.41 - 1.23)	0.35 (0.19 - 0.51)

Average absorbance levels normalized to the reference absorbance value for pCHK2-thr68 in protein lysates of ex vivo irradiated whole blood in the presence or absence of LPS.

Dose	pCHK2-thr68	
Time	24 hr	24 hr
LPS	no	yes
0 Gy	0.19 (0.14 - 0.27)	0.17 (0.12 - 0.24)
2 Gy	0.29 (0.24 - 0.37)	0.18 (0.13 - 0.27)

Average absorbance levels normalized to the reference absorbance value for pCHK2-thr68 in protein lysates of ex vivo irradiated whole blood in the presence or absence of LPS.

	IL-6	TNF-alpha
no LPS	0.01 (0.01 - 0.02)	0.03 (0.02 - 0.03)
LPS	0.24 (0.08 - 1.01)	0.16 (0.03 - 0.41)
